# Granulomatous Secondary Syphilis: A Clinicopathological Diagnostic Framework From Three Cases

**DOI:** 10.1002/ccr3.73514

**Published:** 2026-09-10

**Authors:** Sandesh Shah, Deepika Neupane, Mohan Bhusal, Deeptara Pathak Thapa, Radhika Maharjan, Joshana Shrestha

**Affiliations:** ^1^ Department of Dermatology Nepal Medical College and Teaching Hospital Kathmandu Nepal; ^2^ Department of Prosthodontics B.P Koirala Institute of Health Sciences Dharan Nepal

**Keywords:** benzathine penicillin, clinicopathological correlation, diagnostic framework, granulomatous secondary syphilis, plasma cell‐rich granulomas, resource‐limited setting

## Abstract

Granulomatous inflammation does not rule out secondary syphilis. The diagnosis can be confirmed by identifying plasma cell‐rich, vasculocentric granulomas alongside reactive serology and a positive therapeutic response, even if direct detection of the organism is not possible.

## Graphical abstract

Granulomatous secondary syphilis is a rare clinical and histopathological presentation that may mimic other granulomatous dermatoses. Clinic pathological correlation, appropriate serological testing, and recognition of characteristic histopathological features are essential for accurate diagnosis.

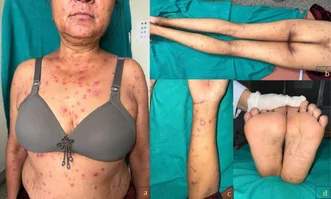

## 1 Introduction

Syphilis, a long‐term multisystem infection caused by *Treponema pallidum* , remains a diagnostic challenge due to its diverse presentations across different disease stages. Although it is both preventable and treatable, its worldwide incidence has increased again over the past decade, driven by changes in sexual behaviors, reduced condom use, and increased online sexual networking [1, 2, 3]. The World Health Organization estimates about 7.1 million new cases annually [2]. Secondary syphilis usually appears as a symmetrical papulosquamous rash affecting the trunk, palms, and soles, often with mucocutaneous lesions and systemic symptoms. Nonetheless, atypical forms, such as the granulomatous variety, are being identified more frequently [1, 4]. Granulomatous secondary syphilis (GSS) is an uncommon histopathological variant of secondary syphilis characterized by epithelioid granulomatous inflammation in the dermis [3, 5]. Recent systematic review evidence has confirmed that granulomatous inflammation represents an infrequent histologic pattern in secondary syphilis, contributing to diagnostic uncertainty and overlap with other granulomatous dermatoses [6].

In settings with limited resources, where techniques such as immunohistochemistry, PCR, or dark‐field microscopy are unavailable for direct detection of the organism, diagnosis relies heavily on clinical features, serology, and characteristic histopathology. This highlights the importance of well‐defined clinical and pathological diagnostic criteria. Because histological features often overlap with other granulomatous diseases, misdiagnosis can occur. This case report aims to highlight the clinical variability and diagnostic challenges of GSS and to emphasize the need for increased suspicion in atypical cases.

## 2 Case Presentations

### 2.1 Case 1

A 55‐year‐old woman presented with a three‐month history of multiple, asymptomatic, progressively enlarging skin lesions on her face, trunk, and limbs, including the palms and soles. She denied any mucosal lesions or constitutional symptoms but reported unprotected heterosexual intercourse a year earlier. There was no history of diabetes, immunosuppressive therapy, systemic illness, prolonged antibiotic use, or previous tuberculosis or leprosy diagnoses. She was not on any regular medications.

**FIGURE 1:**
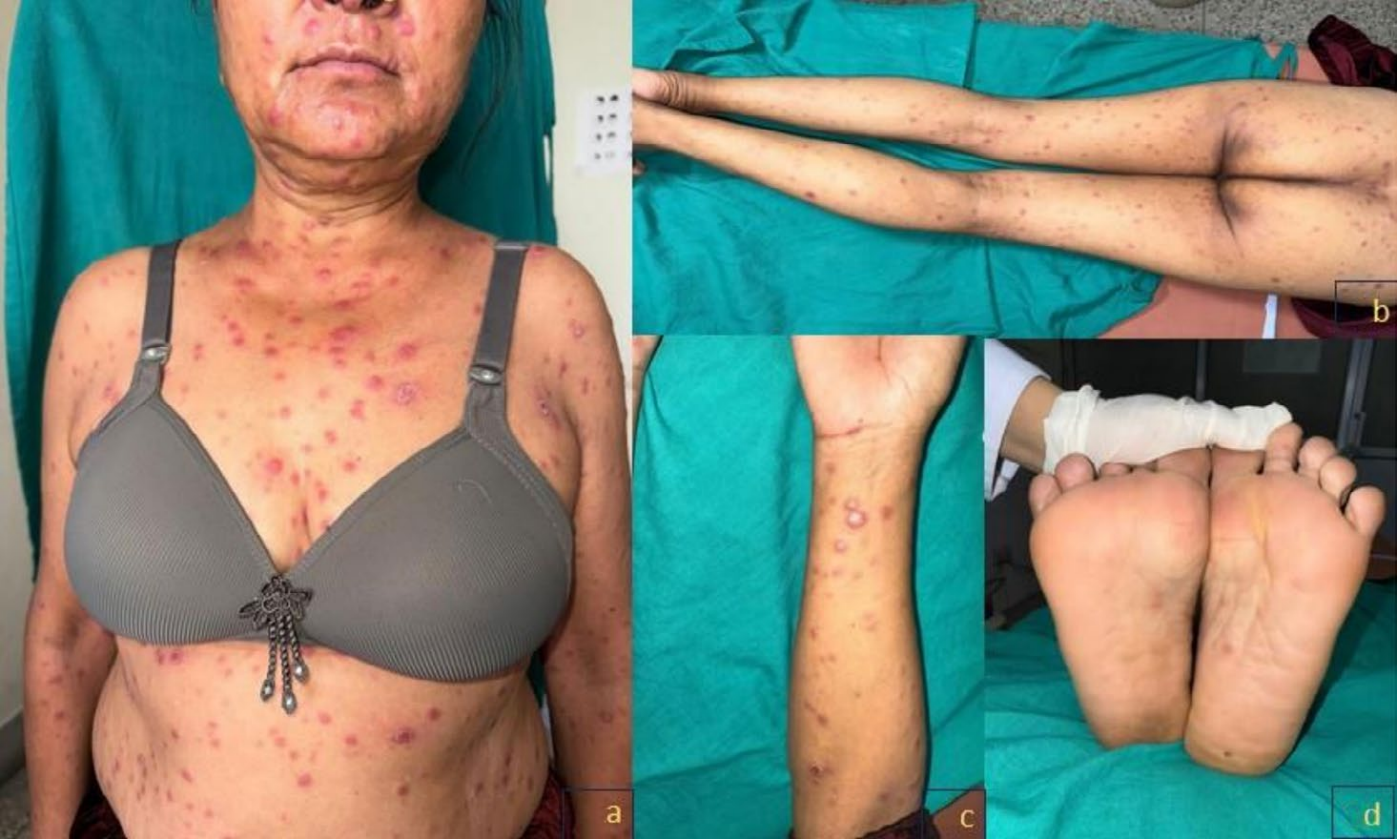
(a–d) (Case 1) Multiple erythematous papules and nodules with fine scaling on the face, trunk, and extremities, including palms and soles.

On examination, numerous erythematous papules and nodules with fine scaling in a collarette pattern were symmetrically distributed over the face, trunk, and extremities, including the palms and soles (Figure 1a–d). Mucosal surfaces appeared normal.

**FIGURE 2:**
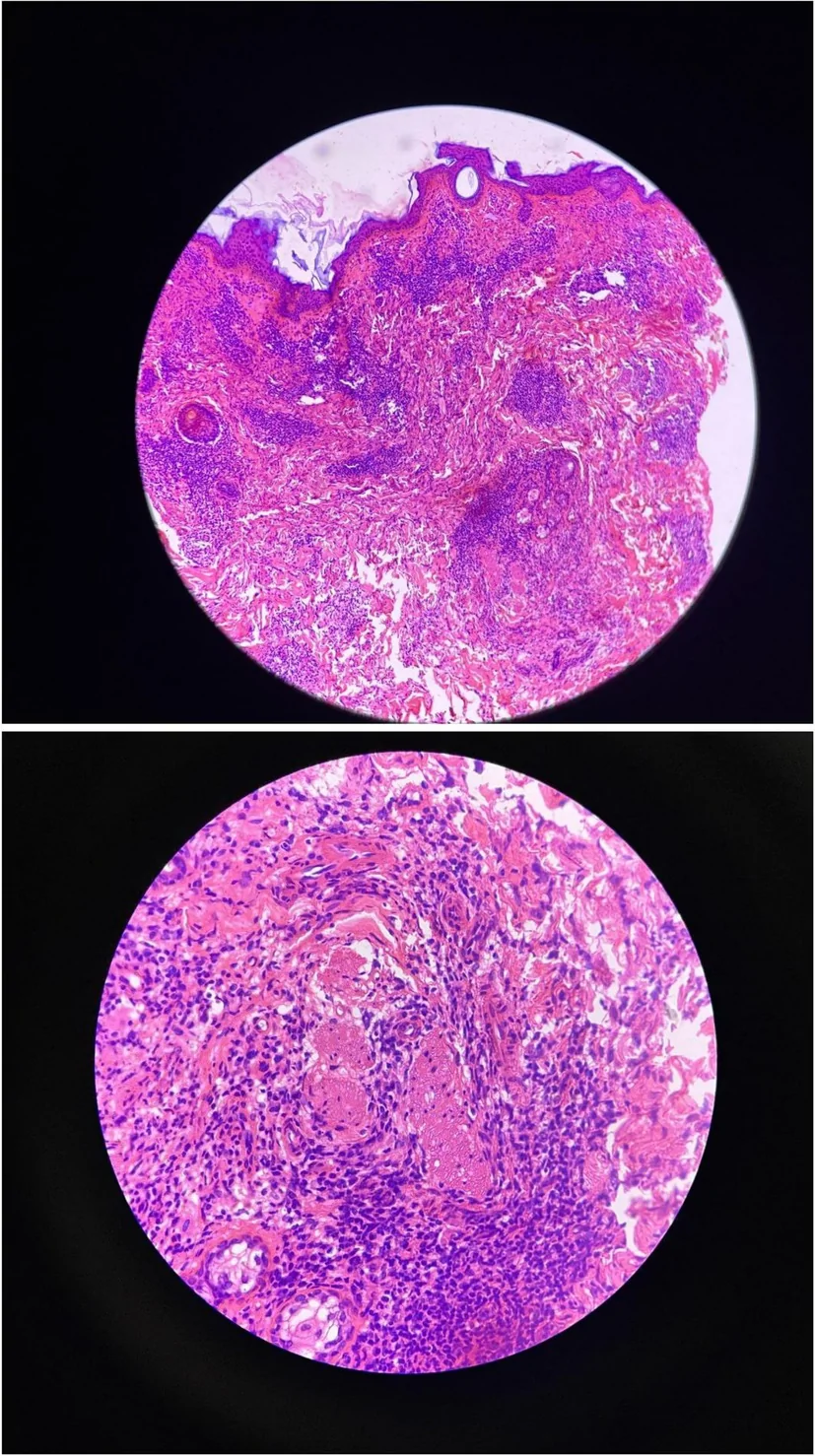
(Case 1) Hematoxylin and Eosin stain showing dermal epithelioid cell granulomas surrounded by dense perivascular lymphohistiocytic and plasma cell infiltrates with endothelial swelling (Upper panel ×4; lower panel ×20).

Laboratory investigations revealed an RPR titer of 1:256 and a positive TPHA test. HIV, HBsAg, and HCV serology were nonreactive. Histopathology revealed noncaseating epithelioid cell granulomas with dense perivascular lymphoplasmacytic and plasma cell infiltrates, as well as endothelial swelling (Figure 2). Stains for microorganisms—PAS, GMS, and Fite—were negative.

The patient was diagnosed with granulomatous secondary syphilis. She was treated with benzathine penicillin G (2.4 million IU IM weekly for 3 weeks), resulting in complete lesion flattening and postinflammatory hyperpigmentation within 6 weeks. At 3 months, the RPR titers had decreased fourfold from 1:256 to 1:32, indicating an adequate serological response.

### 2.2 Case 2

A 43‐year‐old man presented with generalized pruritic papulonodular eruptions lasting 2 months, beginning on the trunk and spreading to the face and limbs. He had a history of unprotected heterosexual contact 9 months earlier. He had no known comorbidities, was not receiving immunosuppressive medications, and denied prior treatment for sexually transmitted infections. There was no history of tuberculosis, leprosy exposure, or recent systemic antibiotic therapy.

**FIGURE 3:**
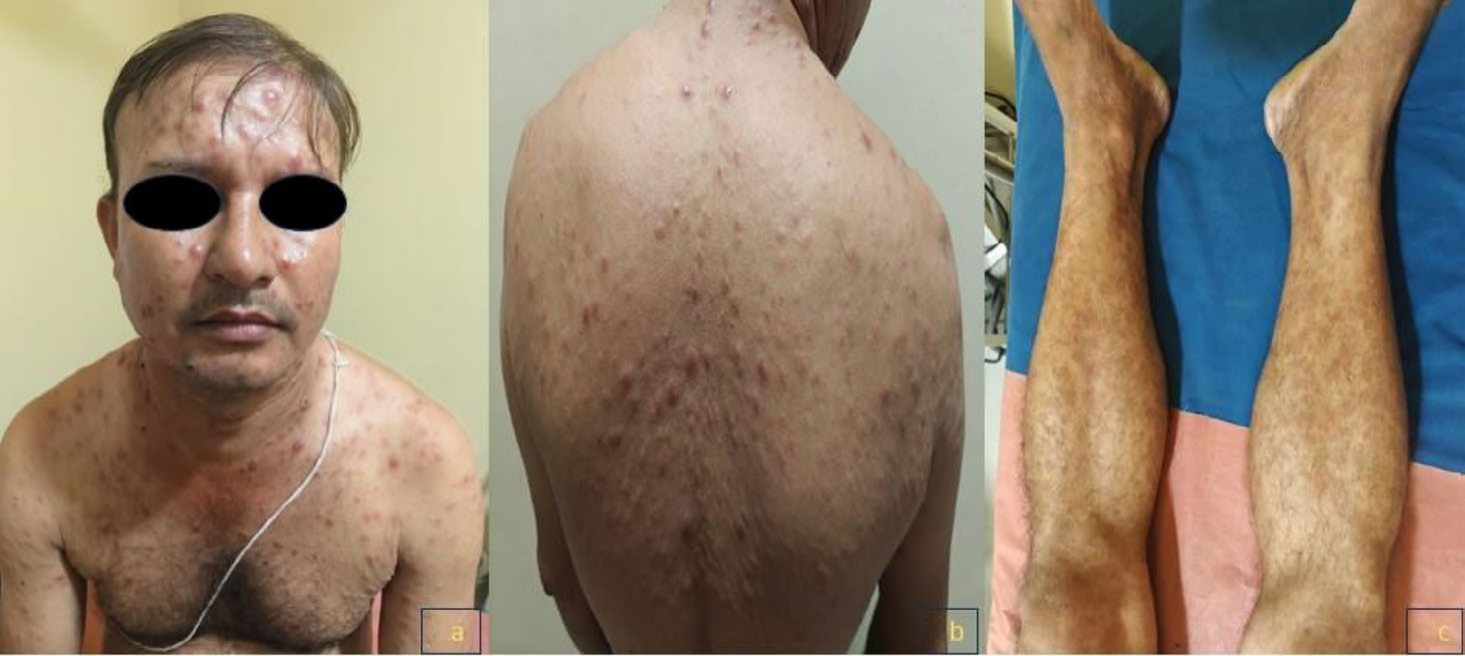
(a–c) (Case 2) Generalized erythematous to hyperpigmented papulonodular lesions over face, trunk, and limbs.

Examination revealed multiple erythematous to hyperpigmented papules and nodules, symmetrically distributed over the trunk, limbs, and face, sparing the palms, soles, and mucosae (Figure 3a–c). No lymphadenopathy was noted.

**FIGURE 4:**
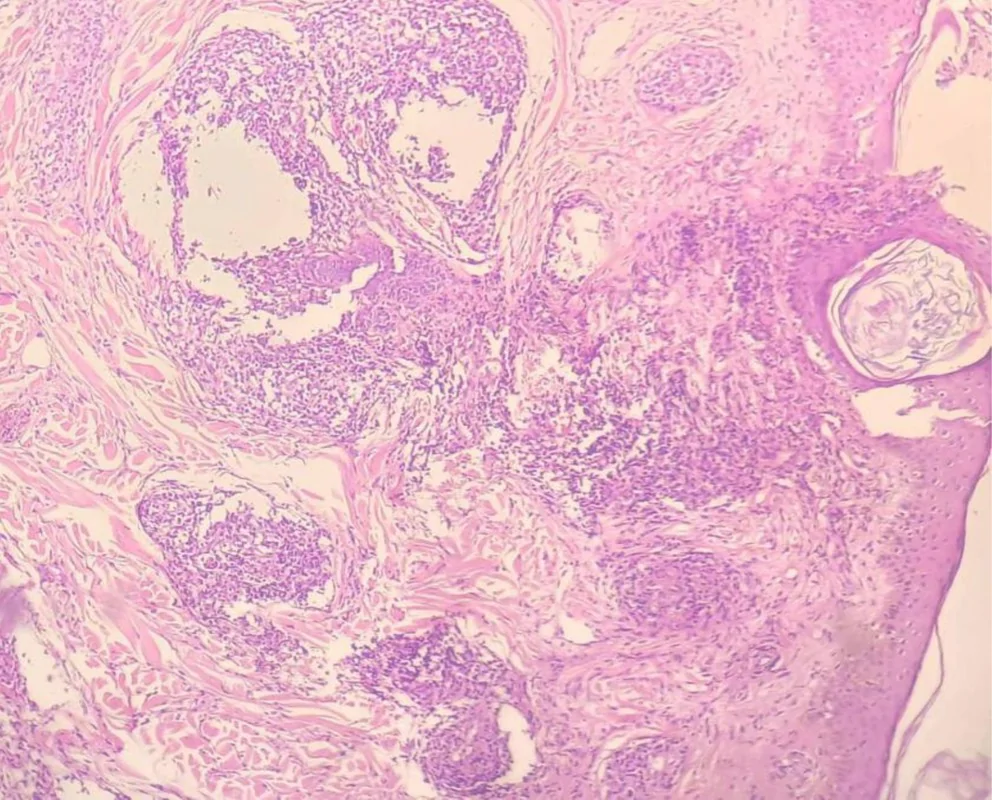
(Case 2) Hematoxylin and Eosin stain (×20) showing dense perivascular and periappendageal lymphohistiocytic infiltrates forming ill‐defined granulomas with numerous plasma cells.

Investigations revealed an RPR titer of 1:512 and a positive TPHA test. HIV, HBsAg, and HCV serology were nonreactive. Histopathology showed dense perivascular and periappendageal lymphohistiocytic infiltrates with ill‐formed epithelioid granulomas and numerous plasma cells (Figure 4). Special stains for AFB and fungi were negative.

After benzathine penicillin G (2.4 million IU IM weekly × 3 weeks), lesions resolved completely within 6 weeks, leaving residual hyperpigmentation. Follow‐up RPR at 3 months showed a decline from 1:512 to 1:64.

### 2.3 Case 3

A 37‐year‐old man presented with asymptomatic, gradually worsening skin lesions over a month. The eruptions started on the trunk and extended to the limbs and face. He reported unprotected sex 6 months earlier but had no genital ulcers or systemic symptoms. He had no history of chronic illness, immunosuppression, past syphilis treatment, or recent antibiotic use. There were no signs of tuberculosis or other granulomatous diseases.

**FIGURE 5:**
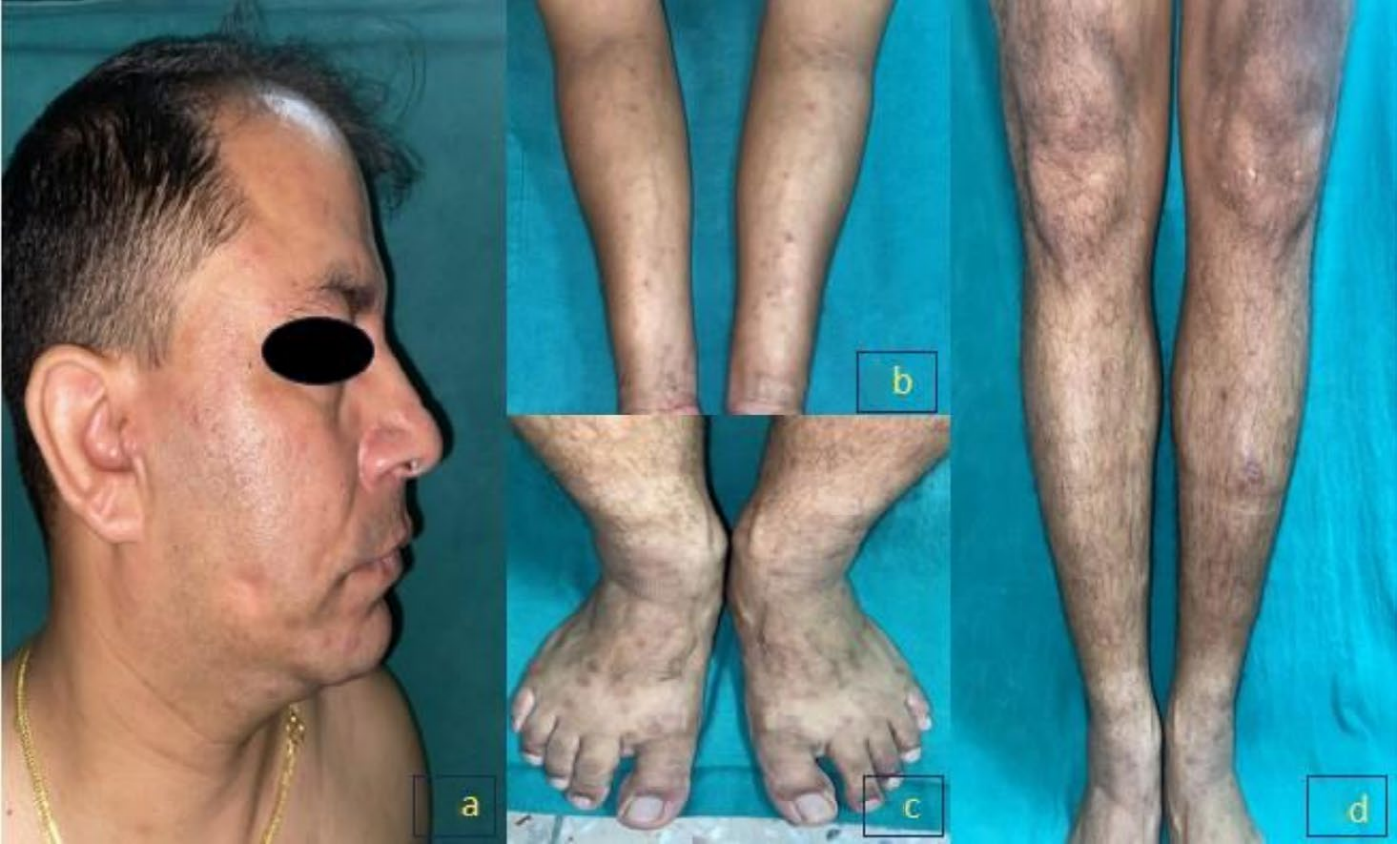
(a–d) (Case 3) Erythematous to violaceous papules coalescing into nodules on the face, trunk, and limbs.

On examination, multiple erythematous to violaceous papules merged into nodules across the trunk, limbs, and face, sparing the palms, soles, and mucous membranes (Figure 5a–d).

**FIGURE 6:**
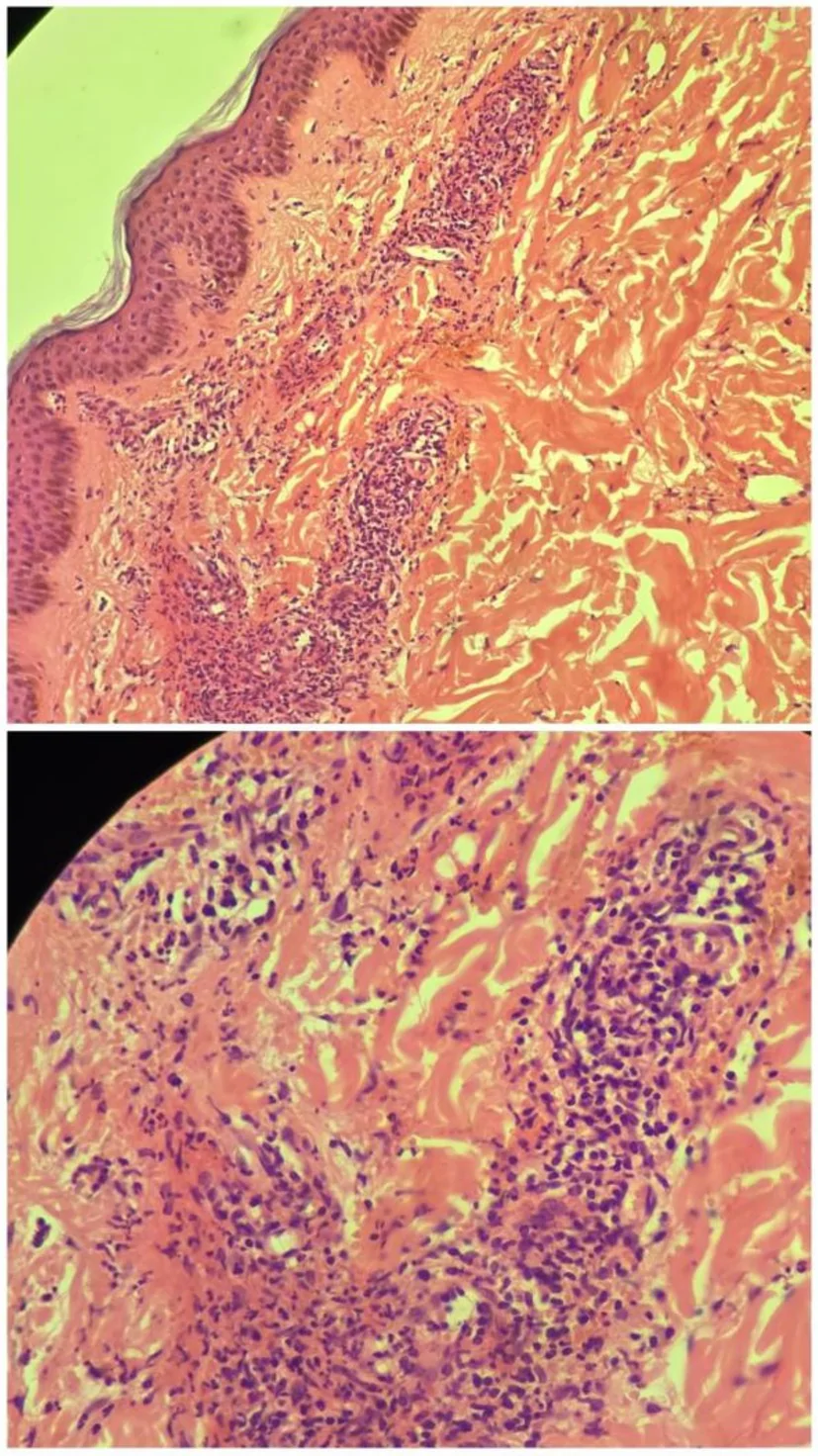
(Case 3): Hematoxylin and Eosin stain showing ill‐formed epithelioid granulomas with prominent plasma cells, lymphocytes, and eosinophils (Upper panel ×10; Lower panel ×20).

Investigations revealed an RPR titer of 1:128 and a positive TPHA test. HIV, HBsAg, and HCV serology were nonreactive. Histopathology revealed hyperkeratosis and spongiosis, with dermal epithelioid granulomas surrounded by lymphocytes, plasma cells, macrophages, and eosinophils (Figure 6). PAS and Fite stains did not detect microorganisms.

Treatment with benzathine penicillin G (2.4 million IU IM weekly for 3 weeks) resulted in complete resolution within 8 weeks. A three‐month follow‐up RPR showed a fourfold decrease from 1:128 to 1:16.

## 3 Discussion

Granulomatous secondary syphilis represents an unusual immunopathological response within the spectrum of secondary syphilis, characterized by granulomatous inflammation due to delayed‐type hypersensitivity to *T. pallidum* antigens [3, 5]. Persistent treponemal antigens stimulate activated CD4+ T lymphocytes and macrophages to produce proinflammatory cytokines such as IFN‐γ and TNF‐α, promoting granuloma formation. This granulomatous reaction occurs in individuals with robust cellular immunity and likely reflects an attempt to contain persistent treponemal antigens rather than direct bacterial invasion [7].

In cases where direct organism detection via immunohistochemistry or dark‐ field microscopy is not available, the diagnosis of granulomatous secondary syphilis was based on a comprehensive clinicopathological assessment: (1) serology showing reactive non‐treponemal and treponemal tests with high titers; (2) clinical features consistent with secondary stage syphilis; (3) plasma cell‐rich granulomatous inflammation accompanied by endothelial swelling; (4) negative special stains ruling out mycobacterial and fungal infections; and (5) full clinical recovery after standard benzathine penicillin treatment. Similar reliance on clinicopathological correlation has been reported previously, especially in settings with limited spirochete detection [3, 8]. Recent evidence further supports this diagnostic approach. In a systematic review and meta‐analysis, Lapenda et al. highlighted endothelial swelling and plasma cell‐rich inflammatory infiltrates as key histopathological features of secondary syphilis across different patterns [6]. These results align with our observations, as all three cases showed prominent plasma cell‐rich granulomatous inflammation and endothelial swelling, even without direct detection of treponemes.

Histopathologically, GSS can closely resemble other granulomatous skin conditions. For example, tuberculoid leprosy shows dense epithelioid granulomas surrounding cutaneous nerves, with AFB positivity on Fite stain. Lupus vulgaris typically presents as caseating granulomas with Langhans giant cells and positive Ziehl–Neelsen staining. Sarcoidosis presents as “naked” granulomas with few lymphocytes and plasma cells—in contrast, deep fungal infections such as histoplasmosis or chromoblastomycosis display identifiable fungal elements on PAS or GMS stains. Granuloma annulare features palisading histiocytes around necrobiotic collagen with mucin deposits. Conversely, granulomatous secondary syphilis shows poorly formed granulomas with abundant plasma cells, perivascular lymphohistiocytic infiltrates, endothelial swelling, and negative special stains [3, 5, 8]. The presence of plasma cells is key to differentiating syphilis from other granulomatous diseases.

Although no prior genital chancre was documented in our patients, primary lesions often resolve spontaneously and may go unnoticed; their absence does not exclude secondary syphilis [2, 5]. Palmoplantar involvement is considered a characteristic feature of secondary syphilis, reported in about 80%–90% of cases [2, 9]. However, some patients may not show lesions on the palms and soles, which can create diagnostic challenges [9]. In our series, two out of three patients did not have palmoplantar involvement, emphasizing the variability in clinical presentation of granulomatous secondary syphilis. Similar absence of palm and sole lesions has also been documented in previous studies of granulomatous secondary syphilis [1, 4, 8].

Our findings align with those of Lim et al., who reported similar cases presenting with generalized lesions, plasma cell‐rich granulomatous inflammation, and an excellent response to benzathine penicillin [1]. Hoang et al. and Kim et al. also described cases that mimicked sarcoidosis and leprosy, underscoring the importance of careful clinical assessment and histopathology [3, 4]. In our series, granulomas were predominantly ill‐formed and distributed in a perivascular and periappendageal pattern, accompanied by dense plasma cell infiltrates and endothelial swelling. This vasculocentric inflammatory pattern has been recognized as a histopathological clue favoring syphilis over sarcoidosis, which typically demonstrates well‐formed “naked” granulomas with minimal surrounding inflammation [3, 4]. The presence of abundant plasma cells further distinguishes syphilis from tuberculoid leprosy and granuloma annulare.

Compared with previously published reports, our series demonstrated consistent serologic follow‐up, with all patients showing a fourfold decline in RPR titers after treatment. In contrast, serologic response was incompletely documented or not clearly reported in several earlier cases [1, 4, 8]. This serologic decline, together with compatible clinical findings, characteristic histopathology, and therapeutic response, further supports the diagnosis of granulomatous secondary syphilis in the absence of direct treponemal detection.

A limitation of our series was the absence of direct detection of *T. pallidum* by immunohistochemistry (IHC), which was not available in our setting. In addition, long‐term serological follow‐up beyond 3 months was not available.

Granulomatous inflammation does not exclude secondary syphilis. In settings where organism detection is unavailable, plasma cell‐rich vasculocentric granulomas, reactive serology, exclusion of alternative infections, and appropriate therapeutic response provide a robust diagnostic foundation. Recognition of this pattern is essential to prevent misdiagnosis and treatment delay.

## Author Contributions

**Sandesh Shah:** conceptualization, methodology, project administration, resources, supervision, writing – review and editing. **Deepika Neupane:** project administration, resources, supervision, writing – review and editing. **Mohan Bhusal:** project administration, resources, supervision, writing – review and editing. **Deeptara Pathak Thapa:** project administration, supervision, writing – review and editing. **Radhika Maharjan:** project administration, supervision, writing – review and editing. **Joshana Shrestha:** conceptualization, methodology, project administration, resources, writing – original draft, writing – review and editing.

## Funding

The authors have nothing to report.

## Consent

Written informed consent was obtained from the patients in accordance with the journal's patient consent policy.

## Conflicts of Interest

The authors declare no conflicts of interest.

## Data Availability

The data supporting the findings of this study are available from the corresponding author upon reasonable request. Patient‐identifying information has been removed to protect confidentiality.
